# An experimental study of foam-oil interactions for nonionic-based binary surfactant systems under high salinity conditions

**DOI:** 10.1038/s41598-024-62610-1

**Published:** 2024-05-28

**Authors:** Ayomikun Bello, Anastasia Ivanova, Denis Bakulin, Timur Yunusov, Alexander Rodionov, Alexander Burukhin, Alexey Cheremisin

**Affiliations:** Center for Petroleum Science and Engineering, Skolkovo Innovation Center, Skolkovo Institute of Science and Technology, 11 Sikorski Street, Moscow, Russia 143026

**Keywords:** Foam, Oil, Interfacial tension, Stability, Binary surfactants, Chemical engineering, Energy infrastructure

## Abstract

A key factor affecting foam stability is the interaction of foam with oil in the reservoir. This work investigates how different types of oil influence the stability of foams generated with binary surfactant systems under a high salinity condition. Foam was generated with binary surfactant systems, one composed of a zwitterionic and a nonionic surfactant, and the other composed of an anionic and a nonionic surfactant. Our results showed that the binary surfactant foams investigated are more tolerant under high salinity conditions and in the presence of oil. This was visually observed in our microscopic analysis and was further attributed to an increase in apparent viscosity achieved with binary surfactant systems, compared to single surfactant foams. To understand the influence of oil on foam stability, we performed a mechanistic study to investigate how these oils interact with foams generated with binary surfactants, focusing on their applicability under high salinity conditions. The generation and stability of foam are linked to the ability of the surfactant system to solubilize oil molecules. Oil droplets that solubilize in the micelles appear to destabilize the foam. However, oils with higher molecular weights are too large to be solubilized in the micelles, hence the molecules will have less ability to be transported out of the foam, so oil seems to stabilize the foam. Finally, we conducted a multivariate analysis to identify the parameters that influenced foam stability in different oil types, using the experimental data from our work. The results showed that the oil molecular weight, interfacial tension between the foaming liquid and the oil, and the spreading coefficient are the most important variables for explaining the variation in the data. By performing a partial least square regression, a linear model was developed based on these most important variables, which can be used to predict foam stability for subsequent experiments under the same conditions as our work.

## Introduction

Foam is widely utilized in EOR operations because of its ability to reduce gas mobility and improve sweep efficiency, thereby enhancing oil recovery from reservoirs^1–3^. The application of foams in EOR is based on their ability to generate a stable gas–liquid dispersion that reduces the viscosity difference between the injection fluid and the reservoir resident fluids, to form a more uniform propagation front for gas mobility control, subsequently leading to an improved oil displacement^4,5^.

Foam stability is a crucial factor in the success of EOR applications, and one of the significant challenges to its application is the interaction between the foam and oil. When in contact with foam, oil can act as an anti-foaming agent by entering thin aqueous films and destabilizing the film. This ultimately results in lower foam viscosity and less stable foams^2,6^. The challenges associated with foam stability are further exacerbated by the high salinity conditions and reservoir heterogeneity of carbonate formations^7–9^, which makes it difficult to maintain foam stability over long periods. Roncoroni et al.^10^ investigated the impact of high salinity on foam stability in carbonate formations, asserting the need for surfactants with improved tolerance to high salinity conditions. Furthermore, predicting the net surface charge of carbonates is a complex task, leading to challenges in controlling surfactant adsorption. Massarweh et al.^11^ explored the difficulties in controlling surfactant adsorption in carbonate reservoirs and emphasized the importance of developing accurate models to predict surfactant behavior under varying reservoir conditions.

Thus, in Bello et al.^12^, we conducted a series of experiments to introduce binary surfactant systems as promising alternatives to single surfactants. When carefully screened and combined, binary surfactant systems have the ability to enhance foaming behavior and stability through synergistic effects, such as increasing solubility, lowering the critical micelle concentration, and improving film elasticity. Jones et al.^13^ asserted that foams generated with binary surfactants tend to have faster foam propagation, higher apparent foam viscosity, and better performance than foams generated with single surfactants because of their enhanced solubility, mobility control, and film stability. By mixing two different surfactants, the solubility of the resulting mixture in both CO_2_ and water can be increased, which can improve foam generation and stability^4^. In AlYousef et al.^1^, a mixture of two nonionic surfactants, one with a longer alkyl chain and one with a shorter alkyl chain, increased the solubility of the surfactant mixture in CO_2_ by reducing the melting point and the critical micelle concentration of the system. The increased solubility can also enhance the adsorption of surfactants at the CO_2_-water interface, which can reduce the interfacial tension and increase the film elasticity.

Nikolov et al.^14^ highlighted the detrimental effects of oil on foam stability and suggested the need for improved surfactant formulations to enhance resistance to oil coalescence and destabilization. Furthermore, Vikingstad et al.^15^ studied the impact of different oil types on foam stability, revealing that light oils tend to destabilize foams more rapidly than heavy and viscous oils.

Oil can interact with foam in different ways. It can enter the foam lamellae, spread on the gas–liquid interface, form a bridge across the foam film, or create a pseudo-emulsion film between the oil and water phases. Thus, several theoretical models have been proposed to describe foam stability in the presence of oil based on these concepts (i.e., entering, spreading, and bridging coefficients and the lamella number)^16^.

The entering coefficient, E quantifies the efficiency of the oil drop entry into the aqueous film and represents the driving force for the oil to enter the foam lamellae^17^.

1 $$\begin{aligned} \ E = \sigma _{w-g} + \sigma _{w-o} - \sigma _{o-g} \ \end{aligned}$$

where: $\sigma _{w-g}$ represents the surface tension between water and gas, $\sigma _{w-g}$ - interfacial tension between oil and gas and $\sigma _{o-g}$ - surface tension between oil and gas.

A positive E indicates that the oil can enter the foam, whereas a negative E implies that the oil is completely wetted by the aqueous phase and cannot penetrate the foam. Bergeron et al.^18^ demonstrates that the entering coefficient is influenced by the interfacial tension between the oil and water. Once the oil enters the aqueous film, its spreading at the gas-water interface becomes a key factor.

The spreading coefficient, S is a measure of the tendency of oil to spread over the gas-water interface^17^.

2 $$\begin{aligned} \ S = \sigma _{w-g} - \sigma _{w-o} - \sigma _{o-g} \ \end{aligned}$$

A positive S value indicates that the oil can spread on the foam, whereas a negative S value implies that the oil forms discrete droplets on the foam surface. Lai et al.^19^ showed that the spreading coefficient was influenced by the viscosity and composition of the oil phase. Understanding the spreading process is crucial for predicting foam stability because a higher spreading coefficient promotes a stable foam. The occurrence of an unstable bridge across the foam film is another mechanism that affects foam stability.

The bridging coefficient, B quantifies the efficiency of bridge formation between neighboring lamellae and represents the stability of the oil bridge that can form across the foam film when the oil-droplet radius is larger than the film thickness^17^.

3 $$\begin{aligned} \ B = (\sigma _{w-g})^2 + (\sigma _{w-o})^2 - (\sigma _{o-g})^2 \ \end{aligned}$$

A positive B indicates that the oil bridge is unstable and can rupture the foam film, whereas a negative B indicates that the oil bridge is stable and can stabilize the foam film. The presence of an unstable bridge can lead to lamellar coalescence and destabilize the foam. Nikolov et al.^14^ showed that the bridging coefficient is influenced by factors such as the surfactant concentration and oil viscosity. Controlling the bridging process is essential for enhancing the foam stability in the presence of oil.

Schramm and Novosad^20^ introduced the concept of lamella number, L as a critical parameter in foam stability.

4 $$\begin{aligned} \ L = 0.15\frac{\sigma _{w-g}}{\sigma _{w-o}} \ \end{aligned}$$

The lamella number represents the degree of oil emulsification in the foam structure and the effect of oil on the film curvature and elasticity. A low L indicates that the oil droplets are small and dispersed in the foam, whereas a high L implies that the oil droplets are large and occupy most of the film volume.

The effects of these coefficients on foam stability in the presence of oil are not straightforward and depend on various factors such as the oil type, surfactant composition, and salinity of the aqueous medium. Thus, several experimental studies have been conducted to investigate the foam stability in the presence of different types of oils, such as crude oil, pure hydrocarbons, lubricating oils, and vegetable oils^2,9,14,15,21,22^. Crude oil has a detrimental effect on foams, and the foam stability decreases as the amount of crude oil increases. However, pure hydrocarbons, such as n-alkanes, have different effects on foams, depending on their chain length and polarity^15^. Short-chain alkanes such as n-pentane and n-hexane have a positive spreading coefficient and can spread on the foam surface, reducing foam stability. Long-chain alkanes, such as n-decane and n-dodecane, have a negative spreading coefficient and form discrete droplets on the foam surface, which can either stabilize or destabilize the foam depending on the bridging coefficient and lamella number^14^.

Previous studies have investigated the foaming behavior of surfactants in the presence of oil; however, to the best of our knowledge, there is no work on the stability of foams generated with binary surfactants under high salinity conditions. Furthermore, from previous studies, there seem to be two factors that are important for foam stability in the presence of oil—the stability of the pseudo-emulsion film and the spreading relation of oil at the water interface. However, there is a lack of understanding of these factors and how they can be used to predict the outcomes of similar experiments.

Therefore, this study aims to address these knowledge gaps by conducting experiments to discuss these issues in detail. Our work is aimed at investigating the stability of CO_2_ foams generated with binary surfactants in the presence of oil under high salinity conditions by conducting stability experiments. Furthermore, foam samples were obtained and visualized under a microscope to better explain the interactions. By conducting drop shape analysis, the IFT of the different fluid systems were measured to compare the oil tolerance using the foaming behaviors of the binary surfactant systems tested. Finally, a multivariate data analysis which includes principal component analysis and partial least regression analysis, was carried our to identify the most important properties influencing foam stability in the presence of oil and suggest a linear model to that can be built on to predict foam stability for subsequent experiments.

## Experimental section

### Materials

Three surfactants were used to perform the experiments, namely: $C_{12}$ AOS (Anionic surfactant, Alpha Olefin Sulfonate, Germany, >99.0 wt% pure), $C_{12}$ Betaine (Zwittterionic surfactant, Cocamidopropyl Betaine, Russia, >99.0 wt% pure), and FARUS (Nonionic surfactant, $C_{2}$ Oxyethylated phenols in monatomic alcohols, Russia,>99.0 wt% pure). In this paper, they are hereafter referred to as AOS, BETAINE and FARUS, respectively. Two binary surfactant systems were prepared from these surfactants: AOS and FARUS, hereafter referred to as AOSFAR, and BETAINE and FARUS, hereafter referred to as BETFAR. All surfactants were used as received without further treatment. The surfactant solutions were prepared in a high salinity brine (23.4 wt%) with the compositions listed in the Table 1 below. All the salt components were purchased from *Chimmed, Moscow* and are >99.0 wt% pure, as declared by the manufacturer.

**Table 1:** Brine composition.

Salt	NaCl	KCl	CaCl_2_	MgCl_2_
Composition, g/L	172.056	4.261	42.172	15.7

The critical micelle concentrations of both single surfactants and their binary mixtures have been earlier determined in our previous work^12^. Thus, in this study, each surfactant was used at 1.5 times their original CMC to maximally harness their enhanced surface activity (Table 2).

**Table 2:** Surfactant compositions and their concentrations.

Surfactant composition	Active content	Concentration, wt%
AOS - Alpha Olefin Sulfonate	35%	0.315%
FARUS - Oxyethylated phenols in monatomic alcohols	50%	0.480%
BETAINE - Cocamidopropyl betaine	75%	0.525%
AOSFAR - AOS + FARUS (1:2)	–	0.135%
BETFAR - BETAINE + FARUS (1:2)	–	0.120%

CO_2_ gas with a purity of 99.98% was used to generate foam. Three mineral oils and one crude oil model were used to investigate the effect of the oil on the foam stability. The mineral oils used were n-Decane, n-Octadecane (liquid paraffin) and Toluene. The crude oil model was a sample from a producing oilfield in Russia. Their densities and molecular weights are shown in Table 3

**Table 3:** Oil properties.

Oil	Density (g/cm^3^)	Molecular weight (g/mol)
Decane	0.83	142.29
Toluene	0.77	92.14
Octadecane	0.88	254.49
Crude oil	0.85	215.74

For mineral oils, 0.05 wt% of Sudan I dye was added to the oil phase to visualize the oil droplets in the foam column. Preliminary tests, including interfacial tension, density and foam columns, were performed to ensure that the dye had no influence on foaming behavior.

### Methodology

#### Foam stability tests

Foam stability in the presence of oil was studied using the standard foaming column setup (Fig. S1, Supplementary information Document). The foaming column is a conventional glass column through which gas is sparged from beneath using a gas diffuser placed at the base of the foaming column.

The implementation of the gas diffuser (80 microns, Saint-Gobain, USA) in this study guarantees a readily and more uniform supply of foaming gas, making it more effective than conventional foam generators and frit glass discs. The foam was generated by sparging 240 ml of gas volume through the surfactant solution of volume 80 ml via the gas diffuser to generate foams of 75% quality. The foam stability was investigated by recording the volume of the foam above the liquid phase as a function of time. 5 ml of oil was added to the surfactant solution before gas sparging. All experiments tests were performed thrice at ambient temperature (23±1^∘^C) and atmospheric pressure.

We also conducted microscopic analyses of some foam compositions. Immediately after foam generation, the foam sample was transferred using a syringe to an electron microscope *(Vinci Technologies, France)* for visualization. The micrographs were captured immediately with no magnifications applied.

#### Interfacial tension using pendant drop analysis

Interfacial tension measurements were performed using the pendant drop shape method^23^ (Fig. S2, Supplementary information Document). Hanging and floating droplets were used depending on the fluids in the system. The interfacial tension was measured by flowing one of the fluids through the capillary into the volume of the other fluid until a droplet was formed and almost separated from the tip of the capillary, as shown in the Supplementary Information Document, Fig. S3.

At this point, an image of the droplet was captured, and the contour of the profile was analyzed using Laplace equation. The principle of operation of the device is based on measurements of the geometric parameters of the image of a liquid drop in a liquid or gaseous medium and image analysis using ImageJ software. The image of the formed droplet was recorded by a video camera, transmitted to a PC, and processed using ImageJ software that outputs a numerical value of the surface tension. Several snapshots (not less than 10) were taken from the video recorded and the surface tension was calculated.

#### Setup for foam viscosity measurement

**Figure 1:**
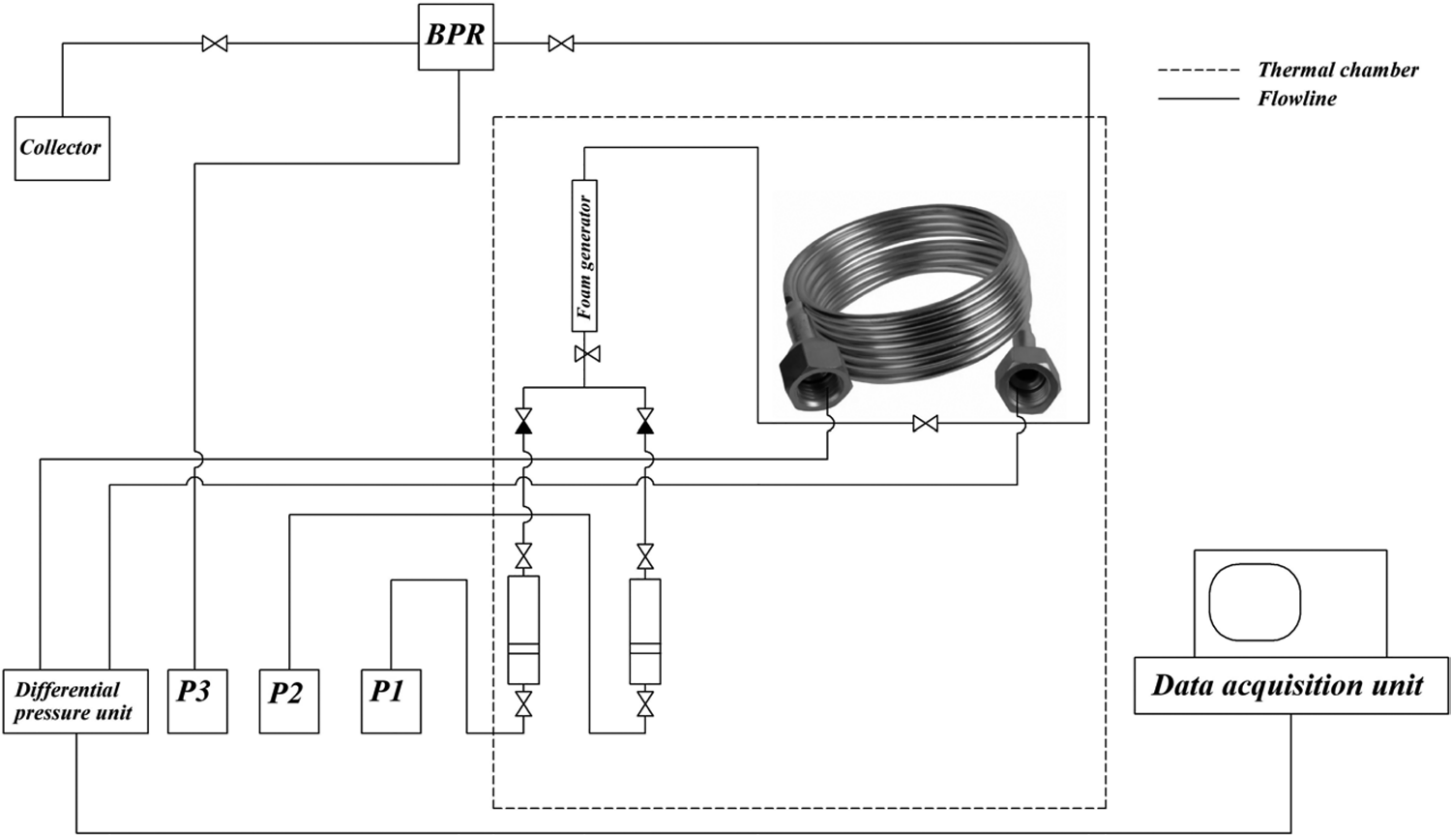
Experimental setup for the measurement of foam apparent viscosity.

The experimental setup used to determine the apparent viscosity of the foam is shown in Fig. 1 below.

The foaming solutions and CO_2_ were injected into the foam generator using two plunger pumps P1 and P2 (GEOLOGIKA, Russia). For each experiment, foam was generated under reservoir conditions of 22 MPa and 41 ^∘^C. The generated foam was allowed to flow through a capillary tube (length = 1200 cm; diameter = 0.51 mm), which was connected to a differential pressure sensor, where the pressure drop between the inlet and outlet of the capillary tube was recorded. During the foam generation and flow experiments, differential pressure data were collected through the data acquisition unit and processed to plot the differential pressure versus time. This allowed us to identify a suitable period during which the differential pressure was relatively constant. The apparent viscosity is calculated using the stable differential pressure based on the Hagen–Poiseuille equation for laminar flow.

#### Multivariate data analysis

We performed a multivariate data analysis to further investigate the parameters influencing foam stability in different oil types. The data consisted of seven input variables, namely molecular weight (MW), interfacial tension (IFT), density, entering coefficient, spreading coefficient, bridging coefficient and lamella number, which are all experimental data obtained in this work for the stability of CO_2_ generated with binary surfactant solutions in the presence of oil.

Firstly, the data was standardized using Eq. (5) to make the input variables comparable and to avoid the influence of scale differences on the analysis results.

5 $$\begin{aligned} \ z = \frac{x - \mu }{\sigma } \ \end{aligned}$$

where $\mu$ is the mean of x and $\sigma$ is the standard deviation of x.

Then, principal component analysis (PCA) was performed on the standardized data using the Eq. (6) to reduce the dimensionality of the data by finding linear combinations of the original variable that capture the most variation in the data.

6 $$\begin{aligned} \ X = USV^T \ \end{aligned}$$

where *X* is the standardized data matrix, *U* is the matrix of scores, S is the diagonal matrix of singular values and V is the matrix of loadings. The PCs are the columns of V.

Thus, a biplot was generated to show the relationship between the different oil types and our experimental data. The biplot was used to detect the parameters that were less important for explaining the variation in the data. Afterwards, a partial least square (PLS) regression was performed to model the relationship between a set of independent variables (X) (the seven input variables) and a dependent variable (y) (the foam height) by extracting latent factors that explain as much of the covariance as possible between X and y. The latent factors were computed by:

7 $$\begin{aligned} \begin{aligned} X&= TP^T + E \\ y&= Tq^T + f \end{aligned} \end{aligned}$$

where X is the standardized data matrix, y is the standardized response vector, T is the matrix of scores, P is the matrix of loadings, q is the vector of weights, E is the matrix of residuals, and f is the vector of residuals. The components are the columns of T.

Thus, a scatter plot was generated to show the actual foam height versus the predicted foam height for each oil type. The plot was used to evaluate the performance of the PLS regression model, which was also measured by the mean squared error (MSE) and the coefficient of determination (R2). A bar plot was also generated to show the loadings of the variables for the first component, which was the most important one for explaining the variation in foam stability.

Finally, linear regression was performed to model the relationship between a dependent variable and one or more independent variables by fitting a linear equation to the data.

8 $$\begin{aligned} \ y = X\beta + e \ \end{aligned}$$

where y is the standardized response vector, X is the standardized data matrix, $\beta$ is the vector of coefficients, and e is the vector of residuals. The intercept is the first element of $\beta$, and the rest are the slopes of the independent variables.

## Results and discussion

### Stability of CO_2_ foams generated with single and binary surfactant systems in deionized water and high salinity brine (23.4 wt%)

In this subsection, we investigated the effect of salinity on CO_2_ foams. The idea is to initially study the behavior and stability of foam in the absence of salinity (i.e. in deionized water). This serves as a baseline for comparison and allows us to effectively assess the ability of the foam to tolerate high salinity conditions (23.4 wt%). Foam stability was assessed by monitoring the foam volume at intervals within 1 h.

The figures below show foam stability in the absence of oil. The goal is to investigate and discuss the salinity tolerance of foams generated with single and binary surfactants.

**Figure 2:**
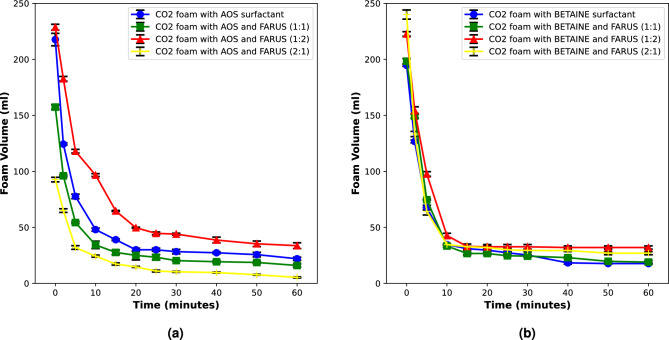
Comparison of the stability of CO_2_ foams generated with surfactant systems without salinity.

**Figure 3:**
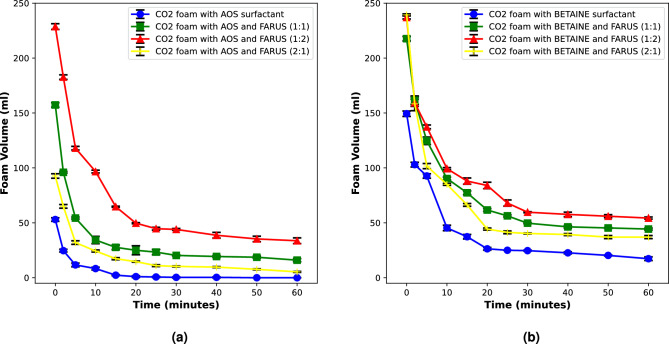
Comparison of the stability of CO_2_ foams generated with surfactant systems with high salinity.

These results in Figs. 2 and 3 show that the foams generated with binary surfactant systems are tolerant to the high salinity condition, as we see that they are stable within the period of 1 h that foam volume was measured (Fig. 3). This shows their potential for utilization in reservoirs characterized by challenging brine conditions. We also see that with the increase in the concentration of non-ionic surfactant, the foam stability increases (Figs. 2 and 3) which supports the results in our previous work^12^ and shows how key the nonionic surfactant component is to the binary surfactant systems.

Indeed, it is also worth noting that not only are the binary surfactant foams more stable, they also generate more foam volume, although the same liquid volume was used in all cases. Also, we should consider that the critical micelle concentrations of binary surfactants are lower than the single surfactants^12^, so this means that even at lesser concentrations and equal volume of gas sparged, the binary surfactant systems still generated more foam volume than the single surfactants. This could be one of the reasons for its increase in foam stability.

In a foam film, the electric double layer plays a crucial role in determining the stability of the foam, especially when there’s a high concentration of electrolytes. The electric double layer is formed at the interface between the foam film and the surrounding aqueous solution. In a foam generated with a single surfactant, the electric double layer is primarily influenced by the charged head groups of the surfactant molecules^24^. Under high salinity conditions, the concentration of ions in the solution is significant. Thus, the increased ionic strength affects the double layer by compressing it due to the screening effect. The ions in the solution partially neutralize the charged head groups of the surfactant, reducing their repulsion and compressing the electric double layer^25^. As a result, the foam film becomes thinner, and the drainage of liquid from the foam film is accelerated. However, the binary surfactant system can lead to the formation of mixed micelles, where the surfactants coexist in a single micelle. This mixed micellar system can have a significant impact on the electric double layer, by modifying the charge distribution at the interface^26,27^. Thus, under high salinity conditions, the binary surfactant system will improve foam stability compared to the single surfactant system (Fig. 3). The interactions between the two surfactants can counteract the compressing effect of ions, leading to a stronger electric double layer^26^. This enhanced stability is attributed to the synergistic effects of the mixed micelles, which can better withstand the challenges posed by high salinity.

The mechanisms underlying the improved stability of binary surfactant-generated CO_2_ foams can further be attributed to the effective hydrophilic–lipophilic balance (HLB) between surfactant molecules at the gas–liquid interface. The synergistic interaction between surfactant components in a binary system allows for a more precise tuning of the HLB, which becomes especially crucial in high salinity environments^28^. A common strategy is to pair a hydrophilic surfactant with a lipophilic one, creating a system that can better adapt to the challenges posed by salinity. Under high salinity conditions, where the aqueous phase is rich in ions, the more hydrophilic surfactant will interact more favourably with water molecules, while the more lipophilic surfactant will preferentially adsorb at the gas–liquid interface, forming a more stable film around the gas bubbles^29^. This selective adsorption and partitioning of surfactants create a dynamic equilibrium, maintaining an effective HLB that resists disruption caused by salinity.

HLB can be calculated using the formula below^30^:

9 $$\begin{aligned} \ HLB = 7 + \sum _{i=1}^{m} H_{i} - n \cdot 0.475 \ \end{aligned}$$

where $H_i$ refers to the $i^{th}$ hydrophilic groups while *m* and *n* refer to the number of hydrophilic and lipophilic groups in the molecule, respectively.

It is assumed that for binary surfactant systems, HLB is additive with respect to the HLB of the individual surfactants^21^. The HLB of a binary surfactant system is computed using Eq. (10) and the corresponding results are presented in Table 4, where it can be seen that, with an increase in the concentration of the non-ionic surfactant, the HLB value of the binary surfactant system was reduced from 39.5 to 19.3 for AOS, and from 29.3 to 15.9 for BETAINE.

10 $$\begin{aligned} \ HLB_{mix} = HLB_A \cdot X_A + HLB_B \cdot X_B \ \end{aligned}$$

where the subscripts A and B refer to emulsifiers A and B, respectively, and $X_A$ and $X_B$ are the mass fractions of the surfactant.

**Table 4:** HLB values of the surfactant systems used in this study.

Surfactant solution	HLB values
AOS	39.5
BETAINE	29.3
FARUS	9.2
AOS + FARUS (2:1)	29.4
AOS + FARUS (1:1)	24.4
AOS + FARUS (1:2)	19.3
BETAINE + FARUS (2:1)	22.6
BETAINE + FARUS (1:1)	19.3
BETAINE + FARUS (1:2)	15.9

Surfactants with low HLB values are more lipophilic and less hydrophilic^4^. Since these experiments were conducted in the absence of oil, this means that they have a greater affinity for the gas phase in the foam and less affinity for the water phase. This means more foam volume is likely to be generated and stability better improved.

**Figure 4:**
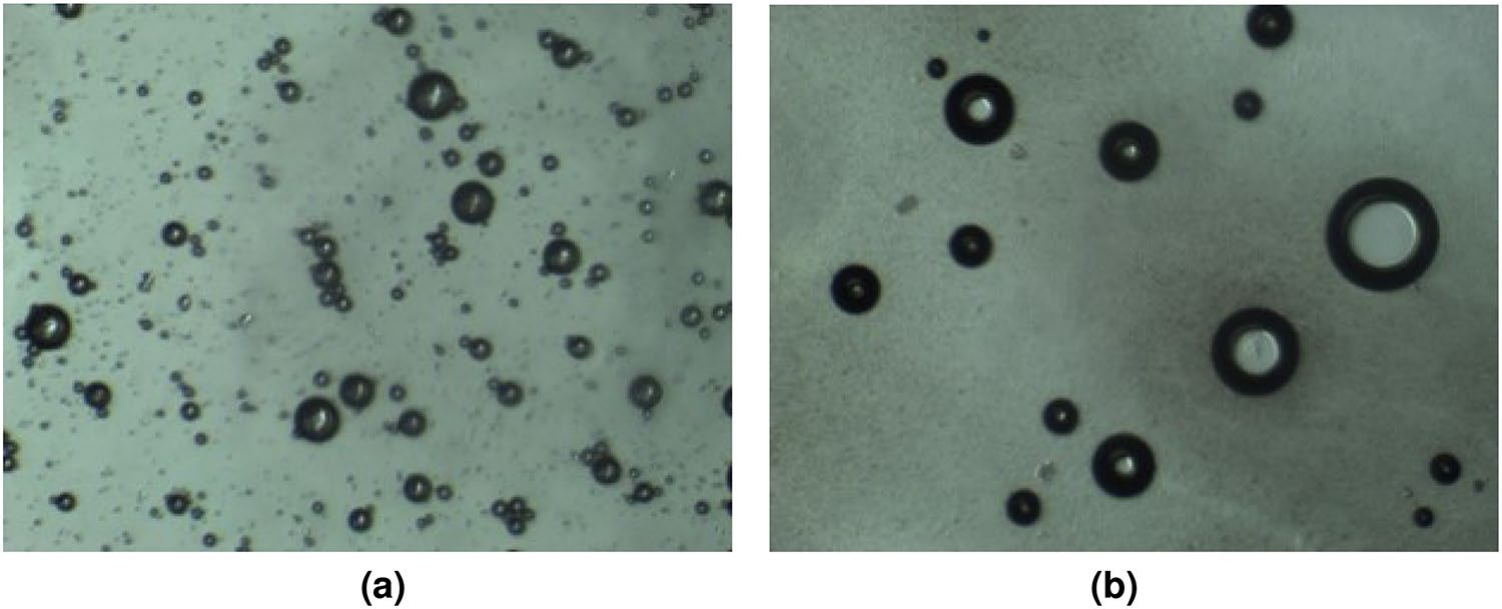
Microscopic images of CO_2_ foam under high salinity condition in the absence of oil.

Additionally, foams generated with binary surfactants can reduce the Ostwald ripening of CO_2_ bubbles better than with single surfactants. In Ostwald ripening, smaller foam bubbles tend to dissolve into the continuous phase while larger ones grow. This occurs because smaller bubbles have a higher curvature, leading to higher internal pressure due to the Laplace pressure difference. As a result, these smaller droplets have a higher chemical potential and tend to dissolve into the continuous phase, transferring their mass to larger foam bubbles^31^. This process can lead to the instability and collapse of foam structures. When binary surfactants are used to generate CO_2_ foams, the synergistic interaction between the surfactants contributes to reducing Ostwald ripening^32^. Binary surfactant systems are designed to have complementary properties that work together to improve foam stability. These surfactant mixtures typically consist of a primary surfactant that stabilizes the foam lamellae and a co-surfactant that assists in reducing the surface tension. This combination leads to the formation of smaller and more uniformly distributed bubbles. The reduced Ostwald ripening in CO_2_ foams generated with binary surfactants can be attributed to the efficient packing of surfactant molecules at the gas–liquid interface^33^. As we see in Fig. 4 below which shows the microscopic images of these foams. These images further corroborate previous explanations.

We can see that the major differences in their structures are the changes in the lamellae thickness and the microstructures of the generated bubbles over time, which are key to foam stability. The binary surfactant system creates a more resistant film around individual bubbles, preventing the mass transfer of material from smaller to larger bubbles. This enhanced stability is particularly beneficial in high salinity environments, where the presence of electrolytes can accelerate Ostwald ripening.

### Influence of oil type on the stability of CO_2_ foams generated with binary surfactant systems in high salinity brine (23.4 wt%)

**Figure 5:**
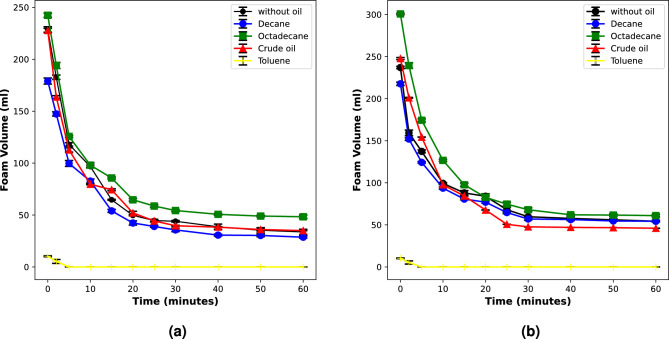
Stability of CO_2_ foam generated with binary surfactants under high salinity condition in the presence of different oil types.

In this section, we discuss the influence of different oil types on foam stability in the presence of oil. The liquid phase was prepared in high salinity brine and CO_2_ foams were generated with binary surfactant systems of AOSFAR and BETFAR. The results are shown in the figure below (Fig. 5).

From these results, we see that in the presence of oil, we could still generate significant volume of stable CO_2_ foam. However, the extent of stability varies. It is also very interesting to see that foam stability is even improved in the presence of octadecane (Fig. 5—green curve) which could be attributed to its molecular weight. The reduced stability of foam in the presence of decane, an alkane oil with a shorter chain length compared to octadecane, aligns well with findings from previous studies. Short-chain alkanes tend to have a destabilizing influence on foams, primarily attributable to their increased solubility in micelles. Koczo et al.^22^ have observed that the solubilization of oil in micelles decreases the effective micellar volume, leading to a reduction in repulsive forces between micelles. This, in turn, causes a step-wise film thinning in foam films, leading to a faster process of foam destabilization. In contrast, longer-chain alkanes, such as octadecane, exhibit a lower tendency to solubilize in micelles, primarily due to steric reasons. In the same vein, Vikingstad et al.^15^ reported that alkanes longer than n-C_10_ do not solubilize in micelles and, instead, contribute to improve foam stability. Furthermore, in Arnaudova et al. (2022) and Denkov et al. (2004)^16,34^ the authors revealed that film plateau borders of the foam are thicker for long-chain alkanes compared to shorter ones. Their results indicate that decane and other short-chain alkanes are distributed both in the foam and in the bulk brine phase, resulting in a higher capacity to solubilize in aggregates, thereby weakening foam stability.

Foam stability in the presence of toluene shows a very poor behaviour (Fig. 5—yellow curve). In both cases, it was difficult to generate foam even during a constant supply of the foaming gas. In the case of BETFAR, when a small volume of foam was generated, there was a cap on that volume and once the gas supply was stopped, there was a rapid coalescence of the foam generated, which suggests that there is a gas saturation limit above which foam cannot be generated. We believe that the inability to generate foam in the presence of toluene can be attributed to several key factors associated with its physical and chemical properties.

Foaming requires the presence of surfactants to reduce the surface tension of the liquid phase and facilitate the formation of stable foam lamellae. However, due to the hydrophobic nature of toluene, surfactant molecules may not effectively adsorb at the air–liquid interface, leading to an inadequate reduction in surface tension and the inability to initiate foam formation^35^. Moreover, toluene is known to dissolve and disrupt the structure of many common surfactants used in foam formation. Surfactant molecules typically organize at the gas–liquid interface to form a stabilizing film around bubbles. However, in the presence of toluene, solubilization of surfactants can occur, leading to the destabilization of the surfactant film. This dissolution prevents the surfactants from effectively reducing the surface tension and forming a cohesive layer at the air–liquid interface, thereby hindering bubble stabilization^36^. Additionally, the volatility of toluene may contribute to the evaporation of any formed bubbles. Toluene has a relatively low boiling point, and the rapid evaporation of toluene molecules from the liquid phase can lead to the destabilization and collapse of the forming foam structures.

As mentioned earlier, the generation of foam involves the formation of a gas–liquid interface. This process requires work, which can be quantified as the product of the interfacial tension/surface tension and the increase in area of the interface. In water, bubbles have a high interfacial energy and become instantly unstable. Therefore, it is essential that surfactant adsorbs at the interface and reduces the surface tension to stabilize the bubbles. The adsorption kinetics plays an important role in the stabilization of the bubble, and the surfactant molecules need to rapidly diffuse from the bulk solution to the bubble interface. However, in the presence of toluene, the situation is different. The surfactant molecules are solubilized almost completely by a strong solvent (Toluene)^37,38^. This could lead to a lack of surfactant molecules at the gas–liquid interface, thereby preventing the reduction of surface tension necessary for bubble stabilization and foam formation.

**Figure 6:**
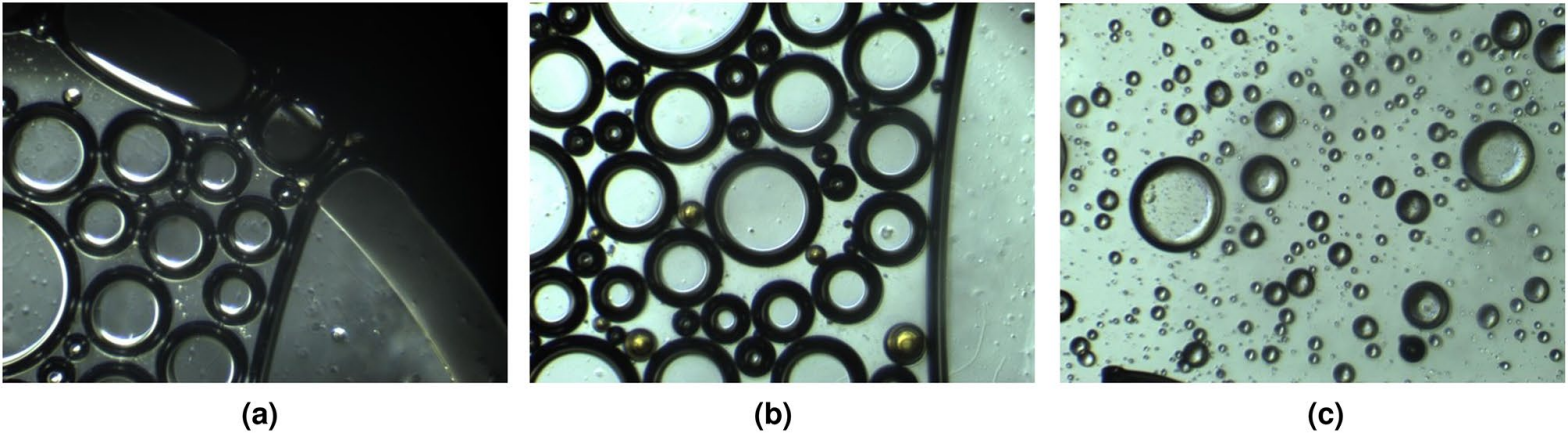
Microscopic images of CO_2_-BETFAR foam under high salinity condition in the presence of different oil types.

Microscopic images (Fig. 6) also support these and give more insights into the explanations as the analyses show a very unstable pseudo-emulsion film in the case of foam generated in the presence of toluene (Fig. 6c). Furthermore, we can also see the orientation of oil droplets in the foam generated. While crude oil has a preference to form large oil globules at the edge of the foam (Fig. 6a), we see the small oil droplets gaining entrance into the foam lamella in the case of foam generated in the presence of octadecane (Fig. 6b). On the other hand, in the presence of toluene, what we see do not seem to look like foam bubbles but rather liquid droplets, which look fragile and less stable, and could explain why foam stability in its presence was very poor.

**Figure 7:**
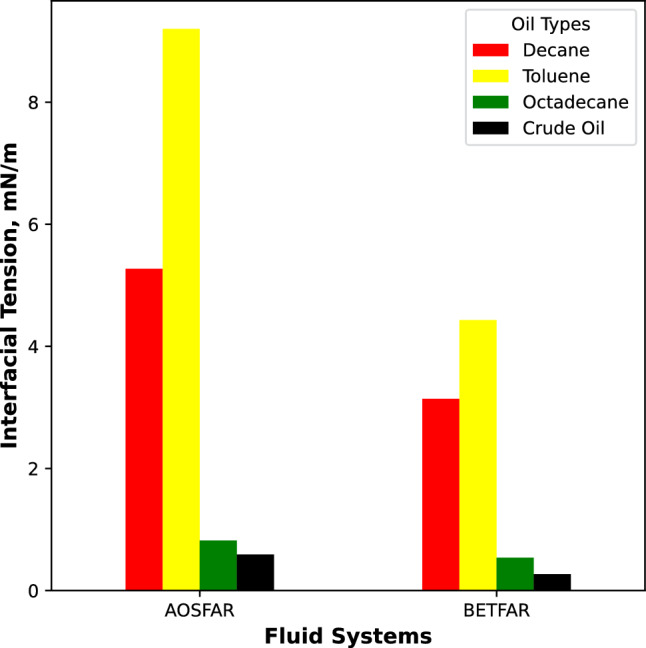
Interfacial tension between the binary surfactant systems and the oil types.

We believe that the emulsification of oil also plays a crucial role in its interaction with foams. When emulsification results in the formation of small oil droplets, they can penetrate the foam structure, influenced by the interfacial tension between the two liquid phases. We measured the interfacial tension for each surfactant mixture in different oil types, and the corresponding values are presented in Fig 7.

Notably, the interfacial tension is lower for BETFAR-Octadecane compared to BETFAR-Decane or BETFAR-Toluene. We hypothesize that a higher interfacial tension between the oil and surfactant leads to the formation of larger oil droplets over time. As the foam dries, the lamellae become thinner, rendering the foam more fragile. Then, due to Ostwald ripening, the average oil droplet size continues to increase, eventually coalescing the foam due to the limited elasticity of the plateau borders. On the other hand, a lower interfacial tension results in slower growth of oil droplets, which remain covered by surfactant molecules^39^. These smaller droplets, with little influence on foam stability, were observed in the foam with octadecane and crude oil, where the liquid phase of the foams exhibited relatively high viscosity. This observation explains the high stability of these foams. However, when other oil types (decane and toluene) were introduced, there was no discernible change in viscosity, which explains why they were rapidly affected by oil. In the subsequent section, we will provide a more detailed explanation of the foaming behaviour mechanism for each binary surfactant system with various oil types.

### Mechanisms of foam-oil interactions: A focus on binary surfactant-generated CO_2_ foams

**Figure 8:**
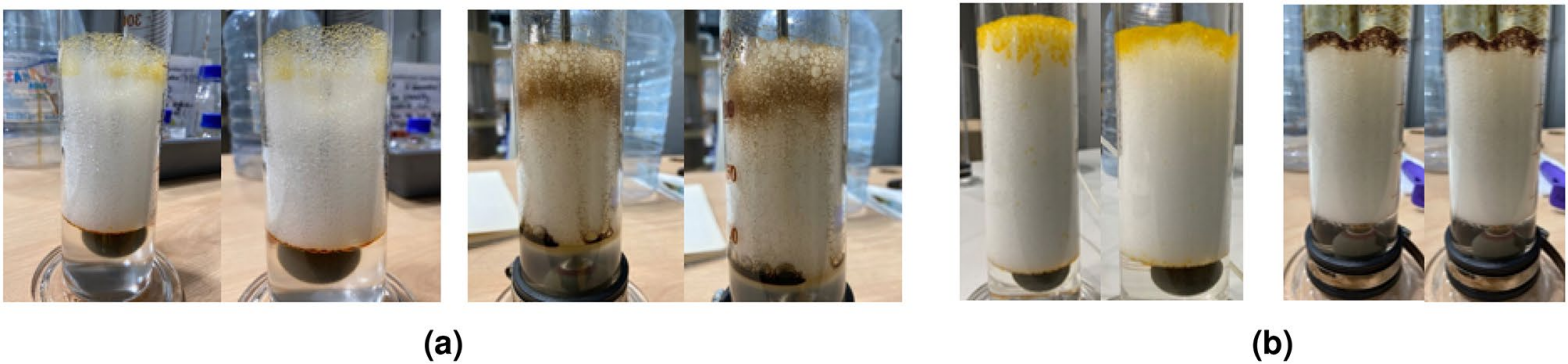
Visual inspection of oil tolerance on the foaming behaviours of binary surfactant systems.

In this section, we analyzed the CO_2_ foams generated with the two binary surfactant systems investigated in the presence of octadecane and crude oil, respectively. Figure 8 below shows the images taken 10 min after generating CO_2_ foams with AOSFAR and BETFAR in the presence of octadecane and crude oil, respectively. From these images, we can compare the oil tolerance between the two binary surfactant systems.

In Fig. 8a, poor mobilization of oil by the foam can be observed, as we can see oil stains around the foam and on the walls of the foaming column (8a, right image). When the propagation front is uniform, only a small volume of oil is mobilized (8a, left image). Contrarily, Fig. 8b shows a uniform propagation front where the majority of the oil in the system is mobilized. These observations suggest a difference in the apparent viscosities of the foam generated by the surfactant systems. Thus, to validate this claim, we conducted experiments to determine the apparent viscosity.

**Figure 9:**
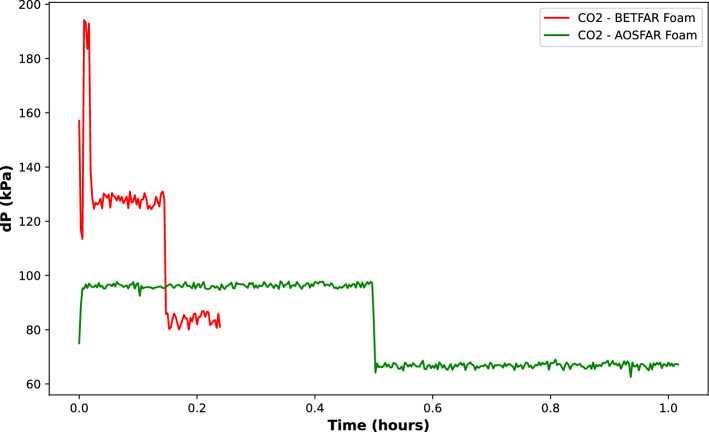
Differential pressure curves of foam flow across a capillary tube.

The pressure drop curves across a capillary tube are presented in Fig. 9. Afterwards, Hagen-Posseuile equation was used to calculate the apparent viscosity and the results are 1.36 cP and 1.75 cP for AOSFAR foam and BETFAR foam, respectively.

11 $$\begin{aligned} \mu _{app} = \frac{\pi \cdot R^4 \cdot \Delta p}{8 \cdot Q \cdot L} \end{aligned}$$

From the results, we see a slight increase in the viscosity of AOSFAR and BETFAR, which could be attributed to the enhanced resistance of the foam films against deformation and rupture. The rigidity of the foam films allows them to withstand external forces, such as shear, more effectively, resulting in higher apparent viscosity values^4^.

**Figure 10:**
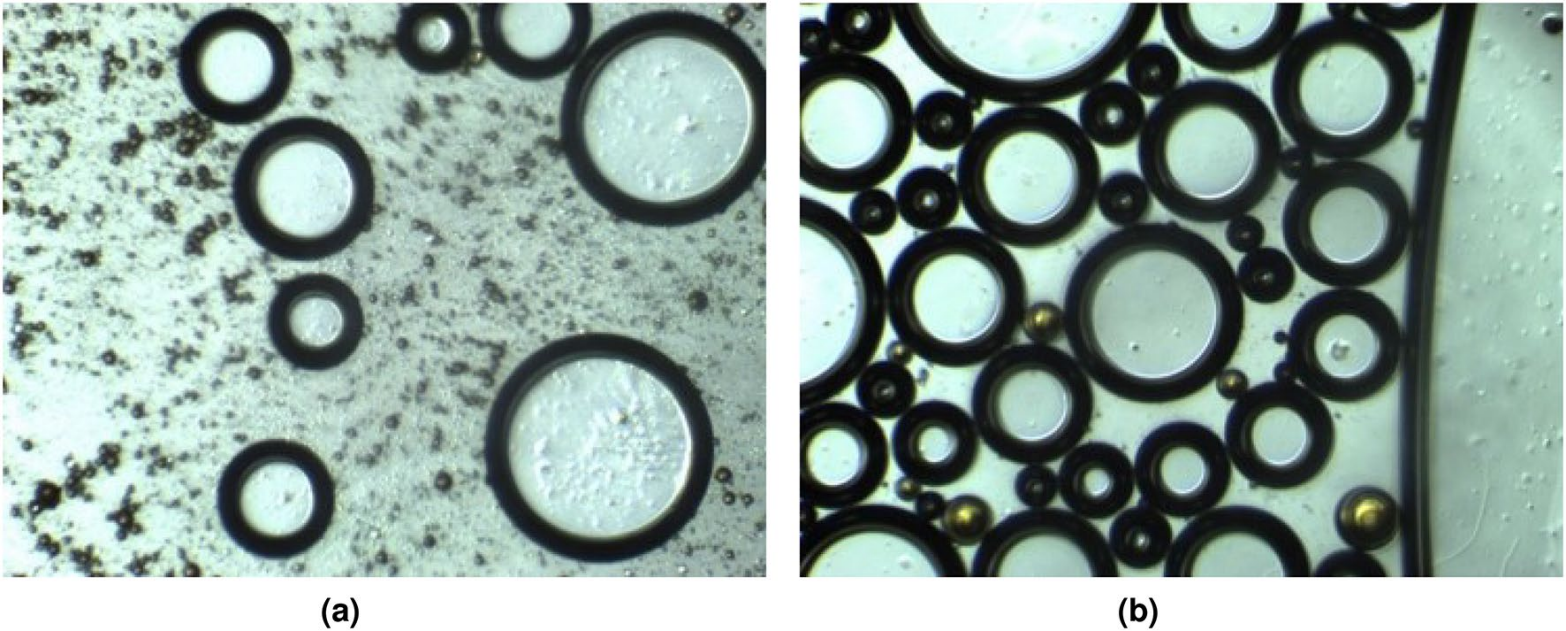
Microscopic inspection of oil tolerance on the foaming behaviours of binary surfactant systems.

Furthermore, from the micrographs in Fig. 10, in AOSFAR, we can observe that for AOSFAR foam, when some oil was mobilized, there’s a high tendency of oil spreading (Fig. 10a) which suggests the possibility of positive entering and spreading coefficients. To provide more insights, we conducted surface tension and IFT tests to validate our findings.

Our investigation of foam-oil interaction began with a visual study, followed by the calculation of various coefficients, as outlined in Eqs. (1)–(4). The corresponding results are presented in the Table 5, showing the coefficients for the tested fluid systems. These values serve as a foundation for explaining the interactions between different oil types and the two binary surfactant systems employed. Consequently, we can build a case for understanding foam-oil interaction mechanisms, particularly in systems utilizing binary surfactants.

**Table 5:** Entering (E), Spreading (S), Bridging coefficients (B) and Lamella number (L) of the oil in the foaming systems in this study.

Oil type	AOSFAR				BETFAR			
	E	S	B	L	E	S	B	L
Decane	14.19	8.23	630.25	1.68	5.60	3.32	218.41	1.57
Toluene	11.72	6.32	527.02	1.85	4.78	4.24	121.78	1.59
Octadecane	8.05	− 6.01	116.35	0.99	− 2.64	− 8.82	− 327.78	0.97
Orenburg	11.71	3.49	65.60	1.22	3.43	− 1.73	51.10	1.54

From the results, we can infer that droplets from all oil types connect to the film surface, enter the lamella (E > 0), and create a bridge between the two liquid interfaces (B > 0). In the case of BETFAR, only decane and toluene exhibit positive spreading coefficients (S > 0), making them prone to spreading on the foam lamella. Conversely, in AOSFAR, only octadecane shows a favourable condition with a negative spreading coefficient. Comparing decane and octadecane, the spreading coefficient is larger for the alkane with a shorter chain length (decane), indicating a higher tendency for the oil phase to destabilize foam. Our results align with Aveyard et al.^40^ where the authors mentioned that the bridging coefficient increases as the alkane chain length decreases. Notably, the presence of decane and toluene results in the largest bridging coefficient, supporting the observation that lower molecular weight oils pose a greater threat to foam stability.

**Figure 11:**
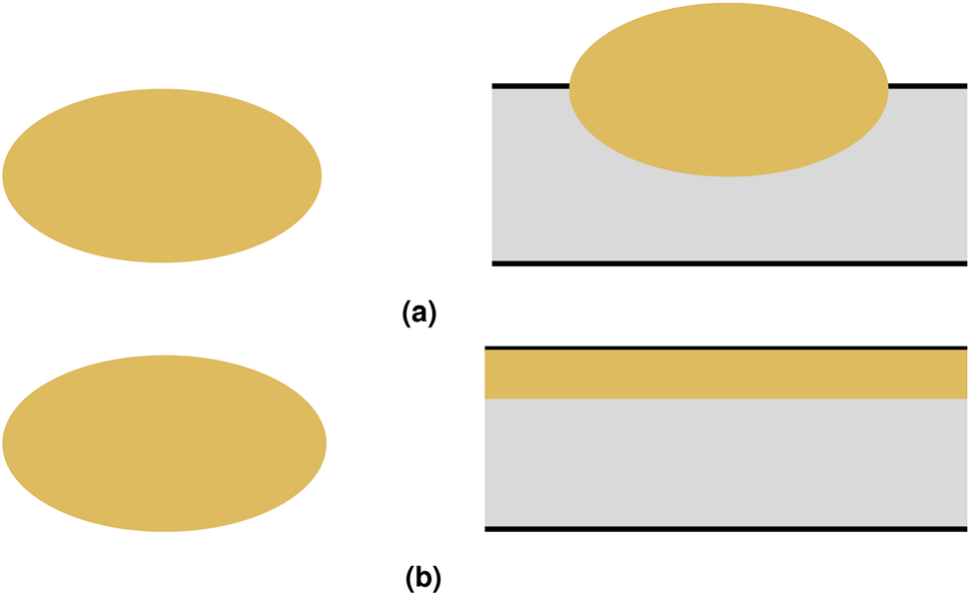
The spreading and non-spreading scenarios of oil into the foam film.

Finally, we observe distinct forms of foam interaction with both crude and mineral oil. It is expected that in cases where the spreading coefficient is positive, the oil will disperse across the film surface and disrupt the foam. On the other hand, when the spreading coefficient is negative, the oil is expected to remain as a droplet at the film surface^14,15,41^. Theoretically, this is considered a prerequisite condition for maintaining stable foam (Fig. 11).

Numerous mechanisms have been proposed to explain the destabilization of foam by oil. Princen et al.^42^ studied an oil droplet on a liquid film stabilized by surfactants at the gas–liquid interface. Experiments, both in the presence and absence of oil, were conducted to show the influence of oil on foam stability. Also, Denkov et al.^16^ studied the oil-induced rupture of foam lamellae. The authors noted that during the initial stages of the drainage process, the presence of oil resulted in the formation of holes in foam films. These studies support our findings, providing a classification of distinctions in the mechanisms governing the interaction between foam and mineral oil versus crude oil.

**Figure 12:**
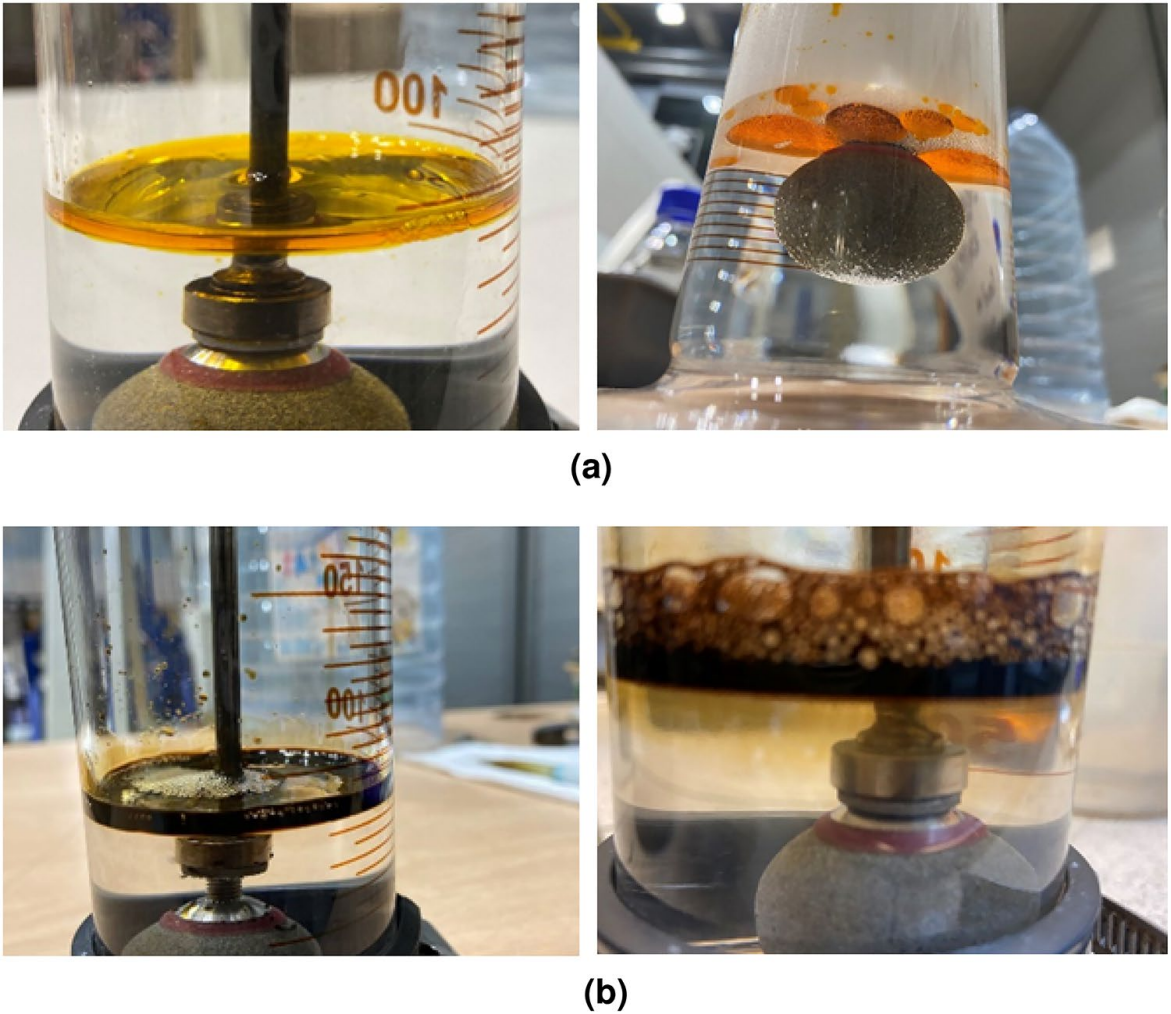
Possible cases of the mechanisms of foam-oil interactions in mineral oil and crude oil.

In the case of mineral oil, represented by octadecane here, we observed a scenario where the oil droplet approaches the gas–liquid interface, covered by a stable aqueous film, and does not enter the foam film; instead, it disperses at the interface (Fig. 12a). In contrast, the crude oil droplet enters the surface and undergoes molecular mixing with the surfactant monolayer, forming a thick film (emulsification), and subsequently spreading as a macroscopic film along the surface (Fig. 12b).

### Multivariate data analysis

**Figure 13:**
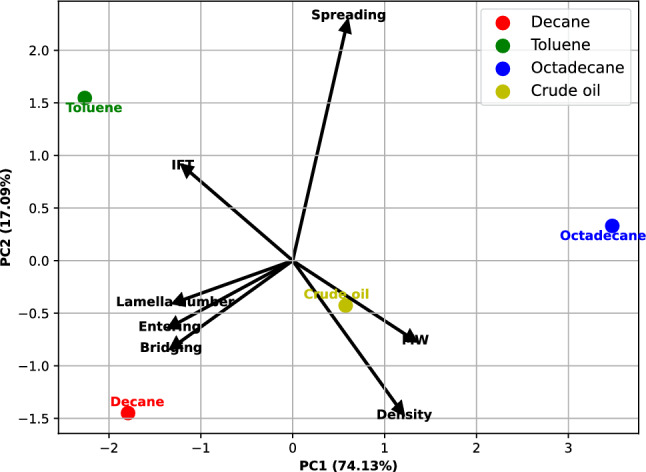
Biplot of the oil data.

In this section, the multivariate data analysis was conducted. The BETFAR binary surfactant system was used for this analysis, since it showed the most favourable results. For the input variables, the entering, spreading, bridging coefficients and lamella number have been earlier shown in Table 5. The density, molecular weight and the interfacial tensions have also been earlier presented Fig. 10 and Table 3. The foam height after 30 min was used as an indicator for stability since there is less gravitational effect impacting foam stability at this point. To obtain the biplot, the PCA analysis was done on the input variables, firstly by standardizing them to have a mean of 0 and standard deviation of 1, after which the covariance matrix of he standardized data was calculated. Then the decomposition on the covariance matrix was performed to obtain the eigenvalues and eigenvectors which represent a principal component and the corresponding amount of variance, respectively. Thus, PC1 and PC2 were chosen to capture the most variance in the dataset. The biplot of the multivariate analysis is shown in Fig. 13

Each point on the biplot represents an oil sample, and the points that are closer to each other have more similar values on the original variables, while the points that are farther apart have more different values. Each arrow represents a variable, and the direction and length indicate the correlation and importance of the variable to the principal components. From the biplot, we can see that the oils are well separated by the first two principal components, which explains about 87% of the total variance.

We can also see the relationship between the objects (different oil types) and the variables (measured data) by looking at the direction and length of the variable vectors. The variables that are closer to the origin and have shorter vectors are less important for explaining the variation in the data. The variables that are farther from the origin and have longer vectors are more important for explaining the variation in the data. The variables that are close to each other and point in the same direction are positively correlated. The variables that are opposite to each other and point in opposite directions are negatively correlated. Thus, we can say that the molecular weight of the oil, density of the oil, interfacial tension between the surfactant solutions and oil, as well as the spreading coefficient of the oil are the most important variables for explaining the variation in the data, as they have the longest vectors and are far from the origin. They are also positively correlated with each other and negatively correlated with other variables.

**Figure 14:**
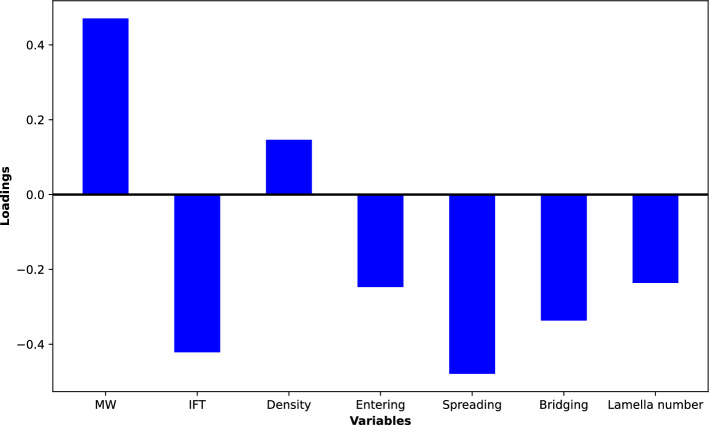
Influence of the input variables on the variations in foam height.

Figure 14 shows the importance of the input variables on foam height. From the bar plot, we can see how different variables explain the variation in foam height. The variables that have higher absolute values of loadings are more important for explaining the variation in foam height. The variables that have positive loadings have a positive effect on foam height, while the variables that have negative loadings have a negative effect on foam height.

We can see that MW, IFT, and the spreading coefficient have the highest absolute loadings. This means that these variables are the most important for explaining the variation in foam height. The other variables have low loadings, which means that they are less important for explaining the variation in foam height. Based on these loadings, we can use MW, IFT, and the spreading coefficient as input parameters to build a linear model that could predict foam height. This confirms the results from the biplot (Fig. 13) as these variables seem to be the most important for foam height.

**Figure 15:**
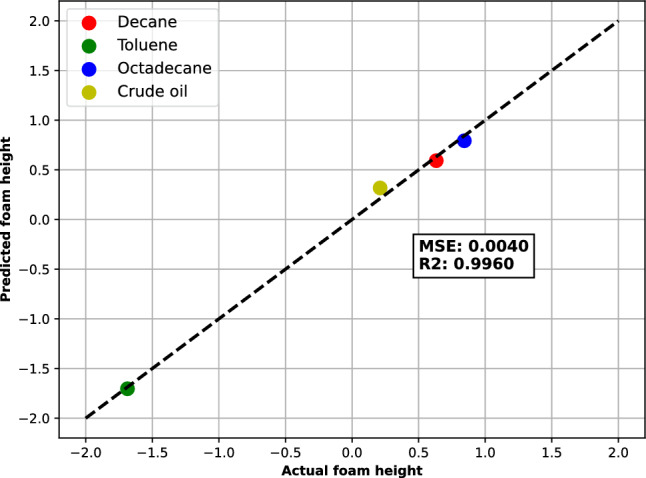
PLS regression of the oil data showing the predicted foam height from the multivariate data analysis.

The plot of the PLS regression results is shown below in Fig. 15 as a scatter plot of the actual foam height versus the predicted foam height with an MSE of 0.0040 and $R^2$ of 0.9960.

The points that are closer to the diagonal line have more accurate predictions, while the points that are farther from the line have more errors. Ideally, we want all the points to be on the line, which means that the model can perfectly predict the foam height for all the oils. The mean squared error (MSE) measures the average squared difference between the actual and predicted values. The lower the MSE, the better the model fits the data. The coefficient of determination ($R^2$) measures the proportion of the variance in the dependent variable that is explained by the model. The higher the $R^2$, the better the model fits the data.

From the plot, we can see that the PLS regression model fits the data well, as the predicted foam height is close to the actual foam height for all the oils. The MSE and $R^2$ values are also very low and high, respectively, indicating a good performance of the model. This suggests that the foam height is strongly related to the other variables in the dataset, upon which the following formula can be defined and means that the linear model can predict the foam height exactly from the three variables.:

12 $$\begin{aligned} \ \text{ Foam } \text{ height } = 5.6\text{ MW } + 3.5\text{ IFT } + 1.8\text{ Spreading } \ \end{aligned}$$

The results of the linear model can be applied to this work and future works, by using the linear model to make predictions of the foam height for new oil samples, given their values of MW, IFT, and the spreading coefficient. The linear model can help estimate the foam stability of different oil types based on their properties, considering that the same conditions used in this work are applied. Furthermore, this linear model can help us to identify the factors that influence the foam height and how they affect it. For example, we can see that an increase in oil molecular weight and a decrease in interfacial tension will increase foam stability in the presence of oil.

## Conclusions

In this work, we studied the interactions between oil and CO_2_ foam generated with binary surfactants for high salinity carbonate reservoirs. We conducted laboratory experiments with 3 mineral oils and a crude oil model from the target reservoir. We performed multivariate data analysis with PCA and PLS regression to examine the factors affecting foam height in different oils. Finally, we developed a linear model that could serve as a basis to build neural models that can be used to predict foam stability based on the most important variables. The main findings of this study include:

- Our results have shown that the binary surfactant foams investigated in our work are more stable and tolerant to high salinity conditions compared to single surfactant foams, demonstrating their potential for application under such harsh conditions. The binary surfactant systems also generated more foam volume than single surfactant, even at lesser concentrations, showing their superiority in terms of stability and efficiency.
- Microscopic analysis of foams shows differences in lamellae thickness and microstructures of bubbles, highlighting a thicker film in the bubbles of foam generated with binary surfactants, preventing mass transfer and enhancing stability in high salinity environments.
- The molecular weight, solubility, and volatility of the oil are the main oil parameters to look out for when foam stability in the presence of oil is concerned, as they affect the surfactant adsorption, solubilization, and film formation at the gas–liquid interface.
- Octadecane enhances foam stability due to its higher molecular weight, resulting in reduced solubility in micelles and thicker plateau borders, while toluene exhibits poor foam stability, attributed to its hydrophobic nature, which hinders surfactant adsorption, disrupts surfactant films, and causes rapid evaporation due to its low boiling point.
- The binary surfactant systems of AOSFAR and BETFAR generated CO_2_ foams with different oil tolerances, with BETFAR showing a more uniform oil front and a higher oil mobilization than AOSFAR which was attributed to an improved apparent viscosity.
- The multivariate data analysis showed that the most important variables for explaining the variation in foam height are molecular weight, interfacial tension, and the spreading coefficient, which have the highest loadings.
- It is important to note that the multivariate data analysis is limited to the conditions and materials tested in this work. However, further studies are required to improve on the linear model in order to build a robust predictive model for the assessment of foam stability.

### Supplementary Information

Supplementary Information.

## Data Availability

The datasets used and/or analysed during the current study available from the corresponding author on reasonable request.
