# Supplementary material for: An experimental study of foam-oil interactions for nonionic-based binary surfactant systems under high salinity conditions
Source: Sci Rep. 2024 May 28;14:12208. doi: 10.1038/s41598-024-62610-1 (PMC11133364; doi:10.1038/s41598-024-62610-1)
Supplement: Supplementary file 1 — Supplementary Information. [file 41598_2024_62610_MOESM1_ESM.pdf]

Supplementary Information Document for: An Experimental Study of Foam-Oil Interactions for Nonionic-based Binary Surfactant Systems under High Salinity Conditions

Ayomikun Bello[a], Anastasia Ivanova[a], Denis Bakulin[a], Timur Yunusov[a], Alexander Rodionov[a], Alexander Burukhin[a], Alexey Cheremisin[a]

[a] *Center for Petroleum Science and Engineering, Skolkovo Institute of Science and Technology, Skolkovo Innovation Center, 11 Sikorski Street,, Moscow, 143026, Russia*

Abstract

A key factor affecting foam stability is the interaction of foam with oil in the reservoir. This work investigates how different types of oil influence the stability of foams generated with binary surfactant systems under a high salinity condition. Foam was generated with binary surfactant systems, one composed of a zwitterionic and a nonionic surfactant, and the other composed of an anionic and a nonionic surfactant. Our results showed that the binary surfactant foams investigated are more tolerant under high salinity conditions and in the presence of oil. This was visually observed in our microscopic analysis and was further attributed to an increase in apparent viscosity achieved with binary surfactant systems, compared to single surfactant foams. To understand the influence of oil on foam stability, we performed a mechanistic study to investigate how these oils interact with foams generated with binary surfactants, focusing on their applicability under high salinity conditions. The generation and stability of foam are linked to the ability of the surfactant system to solubilize oil molecules. Oil droplets that solubilize in the micelles appear to destabilize the foam. However, oils with higher molecular weights are too large to be solubilized in the micelles, hence the molecules will have less ability to be transported out of the foam, so oil seems to stabilize the foam. Finally, we conducted a multivariate analysis to identify the parameters that influenced foam stability in different oil types, using the experimental data from our work. The results showed that the oil molecular weight, interfacial tension between the foaming liquid and the oil, and the spreading coefficient are the most important variables for explaining the variation in the data. By performing a partial least square regression, a linear model was developed based on these most important variables, which can be used to predict foam stability for subsequent experiments under the same conditions as our work.

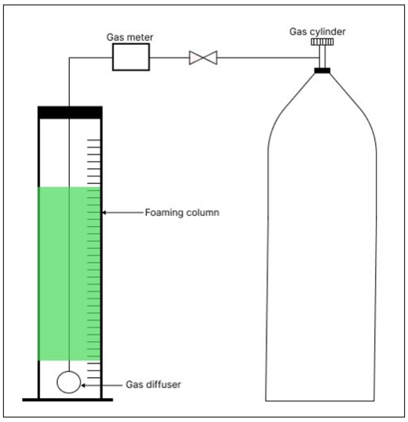

Fig. S1: Experimental setup for foam stability

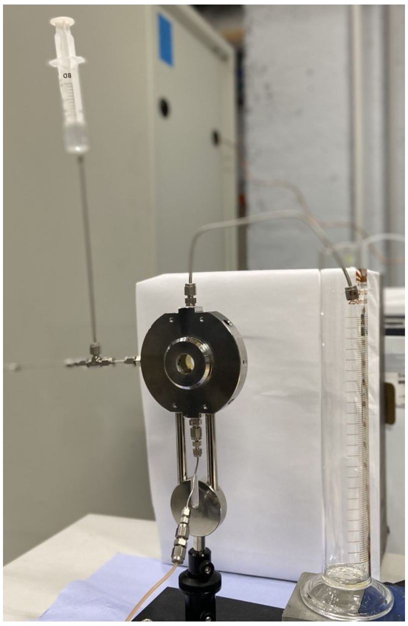

Fig. S2: Experimental setup for IFT measurement using drop shape analysis

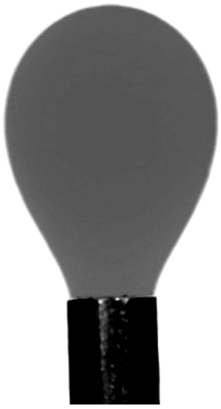

Fig. S3: Image of oil droplet for analysis in ImageJ
